# Deep learning approach to bacterial colony classification

**DOI:** 10.1371/journal.pone.0184554

**Published:** 2017-09-14

**Authors:** Bartosz Zieliński, Anna Plichta, Krzysztof Misztal, Przemysław Spurek, Monika Brzychczy-Włoch, Dorota Ochońska

**Affiliations:** 1 Faculty of Mathematics and Computer Science, Jagiellonian University, 6 Łojasiewicza Street, 30-348 Kraków, Poland; 2 Department of Computer Science, Cracow University of Technology, 24 Warszawska Street, 31-422 Kraków, Poland; 3 Department of Bacteriology, Microbial Ecology and Parasitology, Chair of Microbiology, Jagiellonian University Medical College, 18 Czysta Street, 31-121 Kraków, Poland; 4 Department of Infection Epidemiology, Chair of Microbiology, Faculty of Medicine, Jagiellonian University Medical College, 18 Czysta Street, 31-121 Kraków, Poland; Harbin Institute of Technology Shenzhen Graduate School, CHINA

## Abstract

In microbiology it is diagnostically useful to recognize various genera and species of bacteria. It can be achieved using computer-aided methods, which make the recognition processes more automatic and thus significantly reduce the time necessary for the classification. Moreover, in case of diagnostic uncertainty (the misleading similarity in shape or structure of bacterial cells), such methods can minimize the risk of incorrect recognition. In this article, we apply the state of the art method for texture analysis to classify genera and species of bacteria. This method uses deep Convolutional Neural Networks to obtain image descriptors, which are then encoded and classified with Support Vector Machine or Random Forest. To evaluate this approach and to make it comparable with other approaches, we provide a new dataset of images. DIBaS dataset (Digital Image of Bacterial Species) contains 660 images with 33 different genera and species of bacteria.

## Introduction

The recognition of the bacterial genera and species is crucial since the biological knowledge of microorganisms is extremely important in medicine, veterinary science, biochemistry, food industry or farming. Although most of the microorganisms have positive impact on various areas of life, they can be a reason of many diseases (including the infectious ones). Therefore, automatizing the process of recognition can find application in medical prevention, diagnosis and treatment.

The recognition of microbiological samples is preceded by the culturing process. This phase requires dedicated equipment and chemical agents used for staining the samples. Furthermore, it follows stringent culturing procedures and safety protocols. As the result, we obtain the samples, which are analyzed in order to discover characteristic features and to classify the particular genera and species of bacteria. The classical laboratory methods of bacteria recognition require an expert knowledge and experience. It is a time-consuming process based on comparative analysis of the obtained samples with referential ones (American Bank ATCC; https://www.lgcstandards-atcc.org).

**Fig 1:**
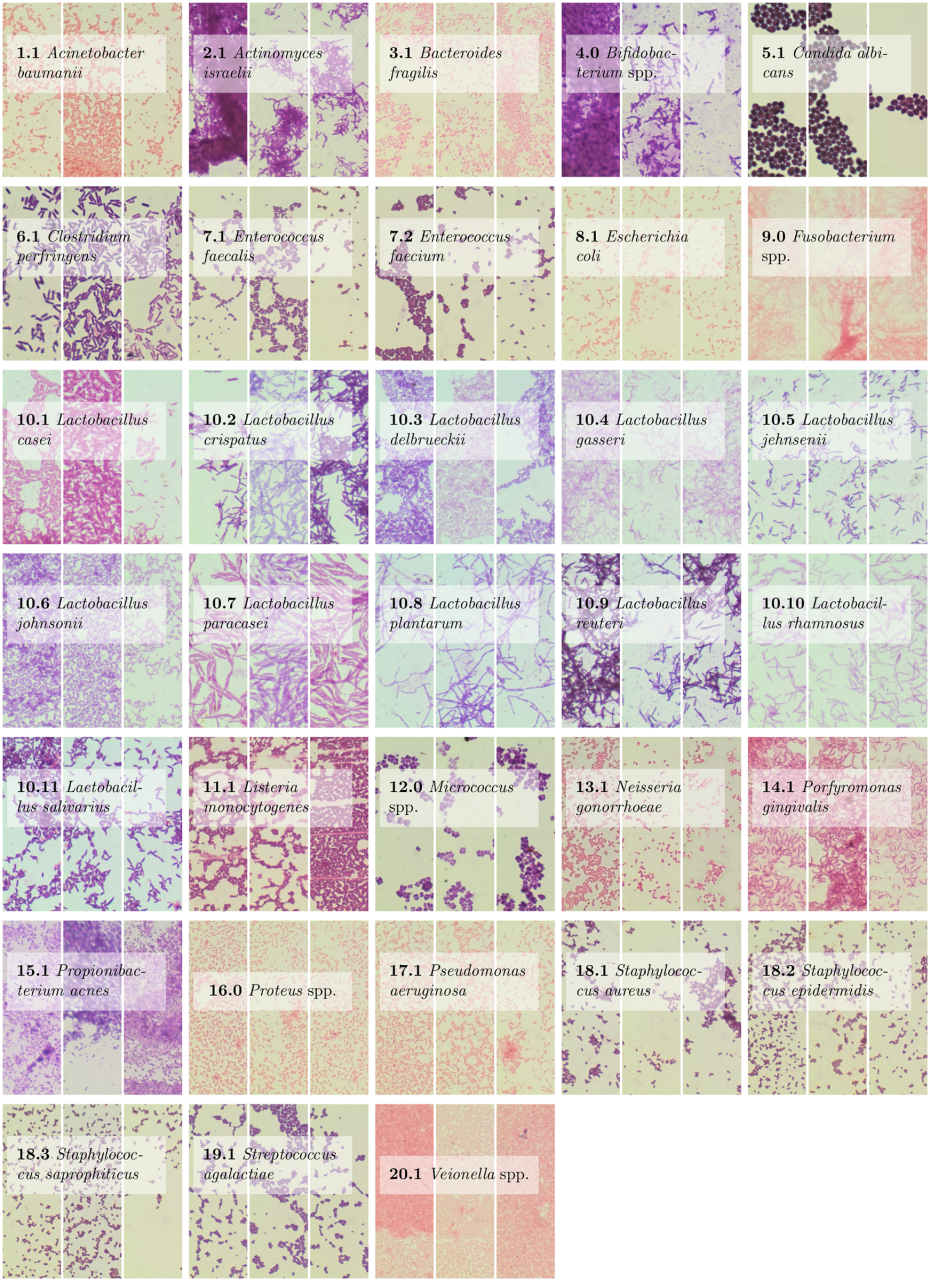
The genera and species of bacteria investigated in this article. There are three examples for each specie to illustrate the significant variability within them.

One of the most important features that can be recognized on the images is the shape of a bacteria cell. We distinguish three basic shapes: cylindrical, spherical and spiral. However, the process of recognizing bacteria based solely on the shape would be a difficult one because many bacteria share very similar shapes. Second most differentiating feature is the shape and the size of the colonies formed by the bacteria. Some of them live solitary, some live in colonies which are very characteristic in terms of structure and spatial arrangement (e.g. resemble longer or shorter chains or letters V, X or Y). However, their shapes can be vastly irregular which also affects the process of recognition. Moreover, some species of bacteria are morphologically diverse and their cells may have multiple sizes and forms. Therefore, the recognition of the bacteria species based on the shape of the bacteria and their colonies is challenging even for experienced specialist and may require additional analysis with the other microbiological characteristics. It can be observed in subfigures 7.1 and 7.2 in Fig 1, which present two different species of very similar bacteria from the same genera. There can also exist a similarities between two different genera (see subfigures 2.1 and 4 in Fig 1).

The purpose of this article is to propose a computer-aided recognition system for classifying bacteria genera and species. For this purpose, we apply the state of the art method for texture recognition introduced by Cimpoi et al. [1]. The choice of this solution is justified by the fact that not only the shape of individual bacteria but also its frequency and the shape of the colony bring important information in the recognition process. This approach allows to solve major disadvantages of the existing methods, which are dedicated to recognize very few species or strains. The main advatage of this method is that it uses Convolutional Neural Network in order to obtain image descriptor. Such descriptor outperforms handcrafted image descriptors, because it contains features learned automatically, based on millions of images [2]. Additional purpose of this project was to provide a new dataset of bacterial images. DIBaS dataset contains 660 images of 33 different genera and species of bacteria. This database is publicly available and can be freely used by the other researchers. The experiments presented in this article can be reproduced with code available online (https://github.com/bartoszzielinski/deep-fbanks). This code was forked from Cimpoi code (https://github.com/mcimpoi/deep-fbanks) and appropriatelly modified in order to cover DIBaS database and new classifiers.

The article is organized as follows. In the next section we review related work on recognizing genera and species of bacteria. After that, we introduce new database of bacterial images called DIBaS, which is publicly available. Section Method shortly describes state of the art method introduced in [1]. The remaining sections contain experiment setup, result and discussion.

## Related works

Image-processing and pattern-recognition techniques combined with various types of classifiers are often used as an effective tools for recognition of the laboratory samples, occurring in the form of images. When taking those methods into consideration, we can state that there are many methods for automatic recognition of bacteria species and strains. There are, among others, statistical methods [3], the artificial neural networks [4–6] or other machine learning classifiers [7].

In [6], an algorithm identifies the species of bacteria based on their geometric features: circularity, compactness, eccentricity, tortuosity and length-to-width ratio. Moreover, because the shape of the bacillus is not a discriminant feature (due to the same morphology in different species of bacteria), it takes into consideration their color.

Ferero et al. [8] describes a method for automated recognition of tuberculosis. It is based not only on geometrical features but also on the average color of the images. In their research, authors tackles the issue of the deceptive similarities in bacterial morphology. He shows that the color of microorganisms is the key feature to improve the accuracy of recognition.

The other approach, presented in [9], applies two classifiers to the pre-segmented scanner images. It uses various measurement features to extract size and shape of the microorganisms and classify them into their appropriate morphotype. The first classifier uses single features to analyze relatively simple communities, containing only a few morphotypes (e.g., regular rods, cocci, and filaments). A second classifier is a hierarchical tree which uses an optimized subset of features to analyze significantly more complex communities, containing greater morphological diversity. Those classifiers automatically categorizes each cell into one of 11 predominant bacterial morphotypes, including cocci, spirals, curved rods, U-shaped rods, regular straight rods, unbranched filaments, ellipsoids, clubs, rods with extended prostheca, rudimentary branched rods, and branched filaments.

Ahmed et al. [10] proposes a method of identification and classification of foodborne pathogens, using colony scatter patterns. In the first step, big set of features are extracted, and then the Fisher’s criterion is used for dimensionality reduction. In the final step, Support Vector Machine (SVM, [11]) classifier is used. Similar approach, with Random Forests instead of SVM, is presented in [12]. Moreover, Ates et al. [13] uses similar approaches to count the number of bacteria colonies, using the compactness ratio of the clusters for the particular species of bacteria. The overview paper concerning those methods was written by Sommer et al. [14].

Most of the described methods are used to recognize very few species or strains (sometimes only one, e.g. tuberculosis). Moreover, in many cases the algorithms base on the morphological features combined with some classification method. Therefore, they are very limited.

The approach used in this article is much more robust and can be used with any genera and species of bacteria.

## DIBaS dataset

Digital Images of Bacteria Species dataset (DIBaS) contains 33 bacteria species with 20 images for each of them. It was collected by the Chair of Microbiology of the Jagiellonian University in Krakow, Poland (http://www.km.cm-uj.krakow.pl/). Table 1 summarizes the genera and species of the bacteria in this dataset while Fig 1 presents fragments of the images.

**Table 1:** The genera and species of bacteria investigated in this article. The ID column present the shorthand name of species used in this article.

GENERA	SPECIES	ID
*Acinetobacter*	*baumanii*	1.1
*Actinomyces*	*israelii*	2.1
*Bacteroides*	*fragilis*	3.1
*Bifidobacterium*	spp.	4.0
*Candida*	*albicans*	5.1
*Clostridium*	*perfringens*	6.1
*Enterococcus*	*faecium*	7.1
	*faecalis*	7.2
*Escherichia*	*coli*	8.1
*Fusobacterium*	spp.	9.0
*Lactobacillus*	*casei*	10.1
	*crispatus*	10.2
	*delbrueckii*	10.3
	*gasseri*	10.4
	*jehnsenii*	10.5
	*johnsonii*	10.6
	*paracasei*	10.7
	*plantarum*	10.8
	*reuteri*	10.9
	*rhamnosus*	10.10
	*salivarius*	10.11
*Listeria*	*monocytogenes*	11.1
*Micrococcus*	spp.	12.0
*Neisseria*	*gonorrhoeae*	13.1
*Porphyromonas*	*gingivalis*	14.1
*Propionibacterium*	*acnes*	15.1
*Proteus*	spp.	16.0
*Pseudomonas*	*aeruginosa*	17.1
*Staphylococcus*	*aureus*	18.1
	*epidermidis*	18.2
	*saprophiticus*	18.3
*Streptococcus*	*agalactiae*	19.1
*Veionella*	spp.	20.0

All of the samples were stained using the Gramm’s method. The images were taken with Olympus CX31 Upright Biological Microscope equipped with a SC30 camera (Olympus Corporation, Japan). They were evaluated using a 100 times objective under oil-immersion (Nikon50, Japan).

DIBaS dataset is publicly available to other researchers (http://misztal.edu.pl/software/databases/dibas/).

## Method

Visual representations based on orderless aggregation of local features, which were originally developed as texture descriptors, have had a widespread influence on the image recognition algorithms. They have been successfully applied to a huge variety of visual domains, including problems closer to “texture understanding” such as material recognition, as well as domains such as object categorization and face identification, despite the fact that on the first glance characteristics of an image of a face shares very little with those of a texture.

In this article, we apply the state of the art texture model to the problem of bacteria species classification. The choice of this method is justified by the fact that different species of bacteria colonies reproduce in a particular manner, resulting in different texture (see Fig 1).

**Fig 2:**
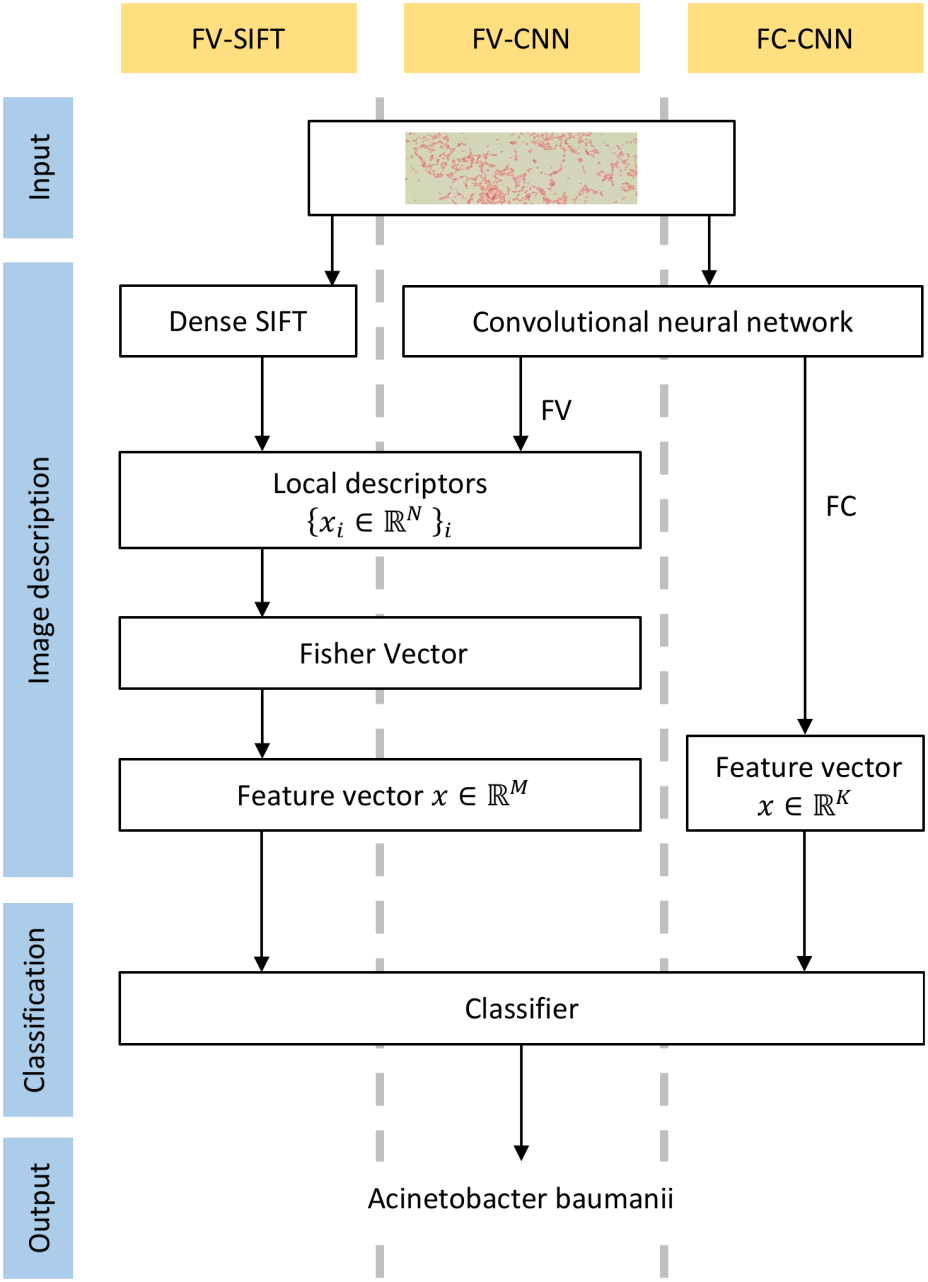
Flowchart of the presented method with successive steps marked in blue. Three image descriptors considered in presented approach are marked in yellow. Two of them (FV-CNN and FC-CNN uses CNN, while the other one (FV-SIFT) uses DSIFT, which is handcrafted descriptor.

We apply the approach proposed by Cimpoi et al. [1], which revisits Fisher Vectors (FV, [15]), a classic texture representation, in the context of deep learning. The successive steps of this approach are as follows: extracting local image descriptors; producing a single feature vector using orderless pooling encoder; and classifying with SVM or Random Forest. The flowchart of this approach is presented in Fig 2. Below, we describe those three steps in details.

*Local image descriptors:* We consider two types of local image descriptors. The first type is the SIFT descriptors [16] extracted densely from the image (DSIFT, [17]). SIFT is the histogram of the image gradients quantized with respect to their location within a patch as well as to their orientation. DSIFT is obtained by sampling with a step of 2 pixels, resulting in *W*/2 × *H*/2 vectors of dimension 128, where *W* and *H* are width and height of the image, respectively. The second type consists of the deep features extracted by truncating Convolutional Neural Network (CNN) at the level of the last convolutional layer. A CNN can be seen as a composition *ϕ*_*K*_ ∘ ⋯ ∘ *ϕ*_2_ ∘ *ϕ*_1_ of *K* functions or layers. The output of each layer *x*_*k*_ = (*ϕ*_*k*_ ∘ ⋯ ∘ *ϕ*_2_ ∘ *ϕ*_1_)(*x*) is a descriptor field $x_{k}\in R^{W_{k}\times H_{k}\times D_{k}}$, where *W*_*k*_ and *H*_*k*_ are the width and height of the layer and *D*_*k*_ is the number of kernels. Therefore, as the result of applying CNN to image *x*, *W*_*k*_ ⋅ *H*_*k*_ vectors of dimension *D*_*k*_ is obtained (assuming that *k*th layer is the last convolutional layer). We consider three CNN architectures: AlexNet [2], VGG-M [18] and VGG-VD [19]. All of them trained on ImageNet dataset (http://www.image-net.org/) [20].

*Pooling encoder:* A pooling encoder takes the local descriptors extracted from an image *x* as an input and produces a single feature vector *ϕ*(*x*_*k*_) as an output. Such vector is suitable for classification, especially with SVM. We use FV, which consists of: computing Gaussian Mixture Model (GMM) for descriptors; assigning descriptors softly to GMM components; and computing first and second order statistics for each GMM component.

*Classifier:* After extracting the FV representation, as set of classifiers is used. One-vs-all linear SVM with *C* = 1 (the classifier originally used by Cimpoi et al. [1]) is called “Original” later in this article. In addition, one-vs-one SVM is investigated with various kernels and their optimal parameters. They are called “Linear SVM”, “Polynomial SVM” and “RBF SVM”, respectively. We use SVM because it has shown stable performance for many important tasks in bioinformatics, such as protein remote homology detection [21], DNA binding protein identification [22], and recombination spot identification [23]. We also use Random Forest (with AdaBoost algorithm) in order to test an ensemble approach. The parameters of the models are computed with Bayes optimization [24].

The representations analyzed in this article are labeled as pairs X-Y, where X is a pooling encoder and Y a local descriptor. For example, FV-SIFT denotes the Fisher vector encoder applied to densely extracted SIFT descriptors, while FV-CNN denotes the same encoder applied on top of the CNN. The possible values of CNN are AN for AlexNet, M for VGG-M and VD for VGG-VD.

We additionally use the output of the last but one fully connected layer (we do not use the last layer, because it corresponds to the classification of 1000 classes from the ImageNet dataset). Although such approach does not use pooling phase, we label it as FC-CNN for consistency (FC in this case stands for fully connected layer).

## Experiment setup

In order to verify if the method dedicated for texture recognition [1] can be successfully applied to classify species of bacteria, we perform two experiments based on DIBaS dataset.

The goal of the first experiment is to verify which of the local image descriptors and pooling encoders results in the best accuracy. For this purpose, the images of each bacterial specie are randomly divided into equally numbered training and test sets. There are 330 images (10 for each bacteria class), both in training and test set. For classification, we use SVM or Random Forest with three types of representations: FV-SIFT, FC-CNN and FV-CNN (see previous Section for details). The experiment is repeated twenty five times in order to obtain stable results. We compute accuracy not only for the particular representations but also for their combinations, as according to Cimpoi et al. [1] they are complementary (e.g. concatenation of FC-M and FV-M is called FCFV-M)

In the second experiment, we examine the scalability of the considered method. We analyze this property, because there exist much more than 33 bacterial species and in the future we plan to extend DIBaS dataset. To predict decrease in accuracy caused by increased number of species, we analyze the accuracy of the method applied to the subsets of our dataset. More precisely, we randomly select *n* out of 33 species, for *n* = {3, 6, 9, …, 31} and train linear SVM with *C* = 1 (the original approach) which classifies only those *n* species. We repeat this procedure 25 times for each *n* in order to obtain smaller variance in results.

## Results and discussion

*The results of the first experiment* are presented in Table 2. It shows that either concatenating FC-M, FV-M, and FV-SIFT, or using FV-M itself give the highest accuracy (sometimes greater than 97%) for all of the classifiers. One can also observe that one-vs-one linear SVM with Bayes optimization works slightly better than one-vs-all linear SVM without optimization, however the difference is not significant. Moreover, polynomial and RBF kernels do not separate classes better than linear kernel, what is probably cause by the small number of training example (only 10 for each class). Such a small amount of training data are also the reason of underfitting in case of Random Forest.

**Table 2:** The accuracy (%) of the presented approaches. Each row corresponds to different descriptor. The first part of the shortcut represents a type of pooling encoder, while the second part is a type of local descriptor. Each column corresponds to different classifier. Original classifier corresponds to linear SVM with *C* = 1 (the classifier originally used by Cimpoi et al. [1]). The other classifiers use Bayes optimization (see Section Method for details). The best results for each classifier are in bold.

Method	Original	SVM			Random Forest
		Linear	Polynomial	RBF	
FV-SIFT	94.58 ±0.68	95.64 ±1.14	94.90 ±1.20	93.70 ±2.92	90.42 ±0.91
FC-AN	82.16 ±1.51	84.96 ±1.12	84.78 ±0.76	84.84 ±1.55	80.60 ±0.42
FV-AN	95.58 ±0.91	96.64 ±0.49	96.28 ±0.45	94.54 ±1.63	91.02 ±1.49
FCFV-AN	94.92 ±0.98	95.52 ±0.98	94.58 ±0.93	93.80 ±1.42	89.26 ±4.23
FCFV-AN & FV-SIFT	96.16 ±0.65	96.34 ±0.93	95.92 ±0.62	94.66 ±1.40	91.56 ±0.75
FC-M	81.32 ±1.25	85.36 ±1.42	84.84 ±2.23	83.58 ±2.94	82.88 ±1.05
FV-M	96.52 ±1.17	**97.24** ±1.07	96.64 ±0.78	**96.52** ±1.15	92.24 ±1.80
FCFV-M	96.46 ±0.80	96.94 ±0.96	96.70 ±0.79	95.96 ±1.33	91.92 ±0.50
FCFV-M & FV-SIFT	**97.06** ±1.03	97.06 ±1.03	**97.00** ±0.99	96.46 ±0.68	92.64 ±0.65
FC-VD	80.12 ±1.66	83.74 ±1.76	84.12 ±1.68	83.68 ±1.18	81.08 ±1.93
FV-VD	96.22 ±0.72	96.58 ±1.01	96.38 ±1.01	95.42 ±0.93	**92.94** ±0.93
FCFV-VD	96.10 ±0.56	96.58 ±0.81	96.22 ±1.05	96.02 ±0.89	92.76 ±0.98
FCFV-VD & FV-SIFT	96.52 ±1.01	96.64 ±1.11	96.30 ±1.78	95.70 ±1.74	92.50 ±0.92

Based on the results of the first experiment, we select the following three methods of feature extraction for the further analysis:

- FV-SIFT,
- FV-M,
- FCFV-M & FV-SIFT,

which are ordered from the least effective (with the simplest architecture) to the most effective (with the most complex architecture).

**Fig 3:**
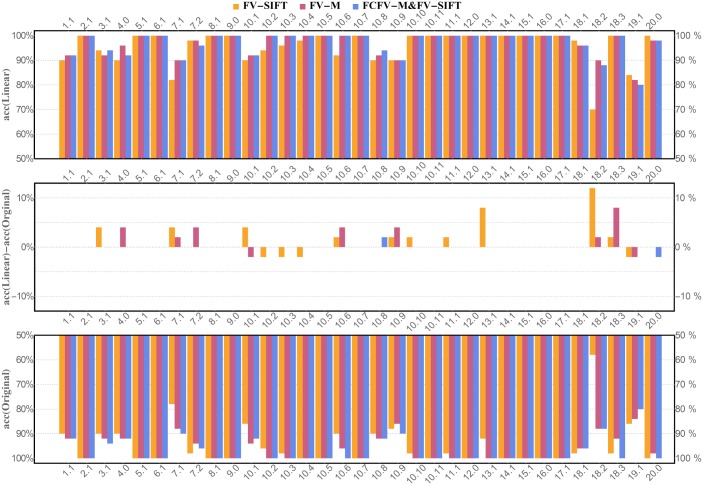
Per class accuracy for three investigated methods: FV-SIFT, FV-M, FCFV-M & FV-SIFT. We compare the results obtained for Linear SVM with optimized parameter (see upper chart) to linear SVM without optimization (see lower chart). The chart in the middle corresponds to difference between both classifiers.

**Fig 4:**
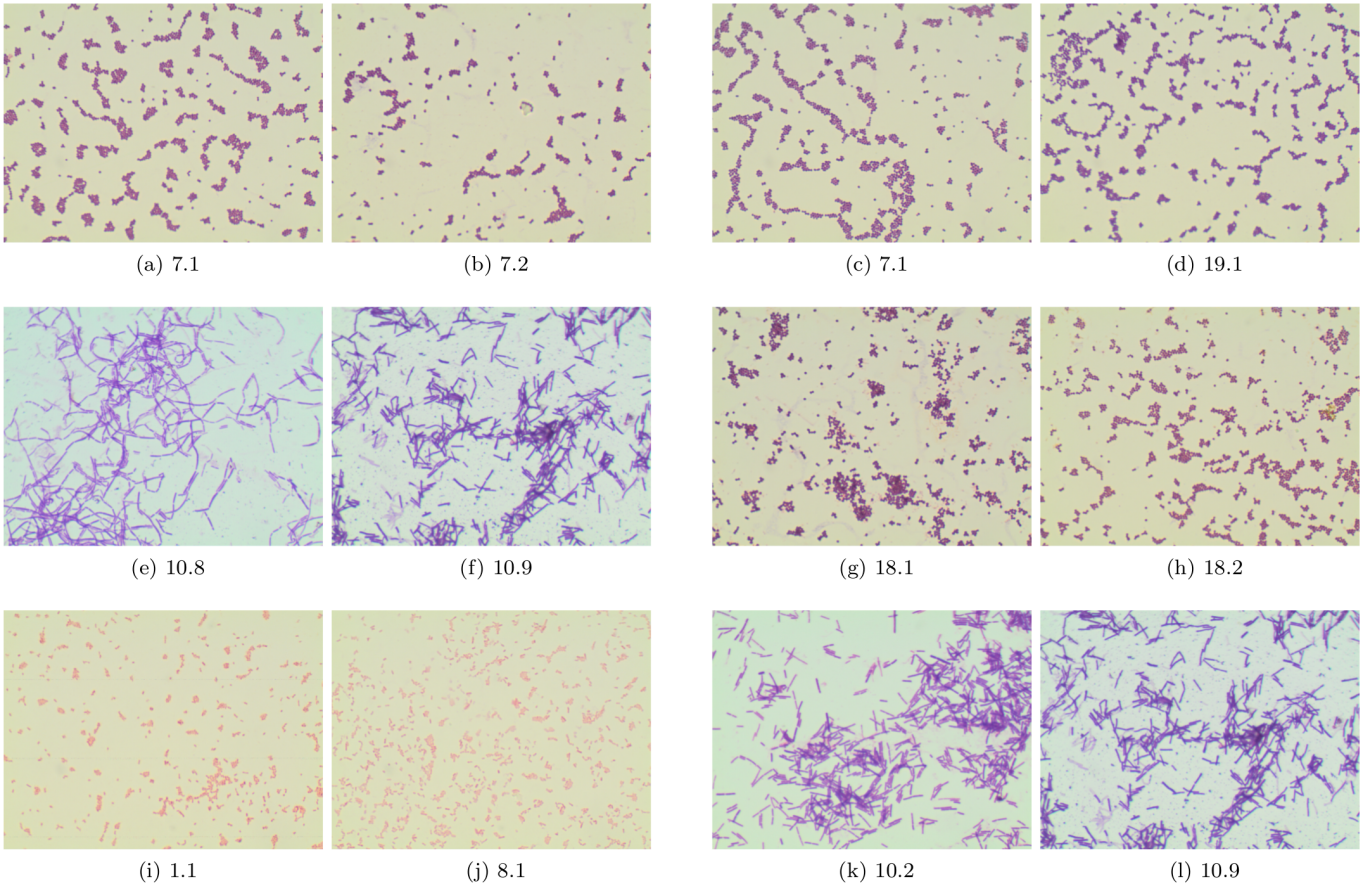
Pair of images representing the random representation of output genera or specie (left image in pair) and the misclassified image (right image in pair).

Per class accuracy for Linear SVM and Original classifier is presented in Fig 3. It can be noticed that the lowest accuracy is obtained for genera and species labeled with 7.1, 18.2, and 19.1 in Fig 1. The examples of incorrect recognitions are presented in Fig 4. It can be observed that they are usually caused by the similarity in bacteria morphology. In particular, genera and species labeled with 7.1, 18.2 and 19.1 may look very similar in the spatial layout in the microscopic image, since all of them represent spherical bacteria that can appear individually or in characteristic post-division arrangements whose shape is related to the plane and number of cell divisions during the propagation. This is probably why, they can be sometimes misinterpreted by the program.

**Fig 5:**
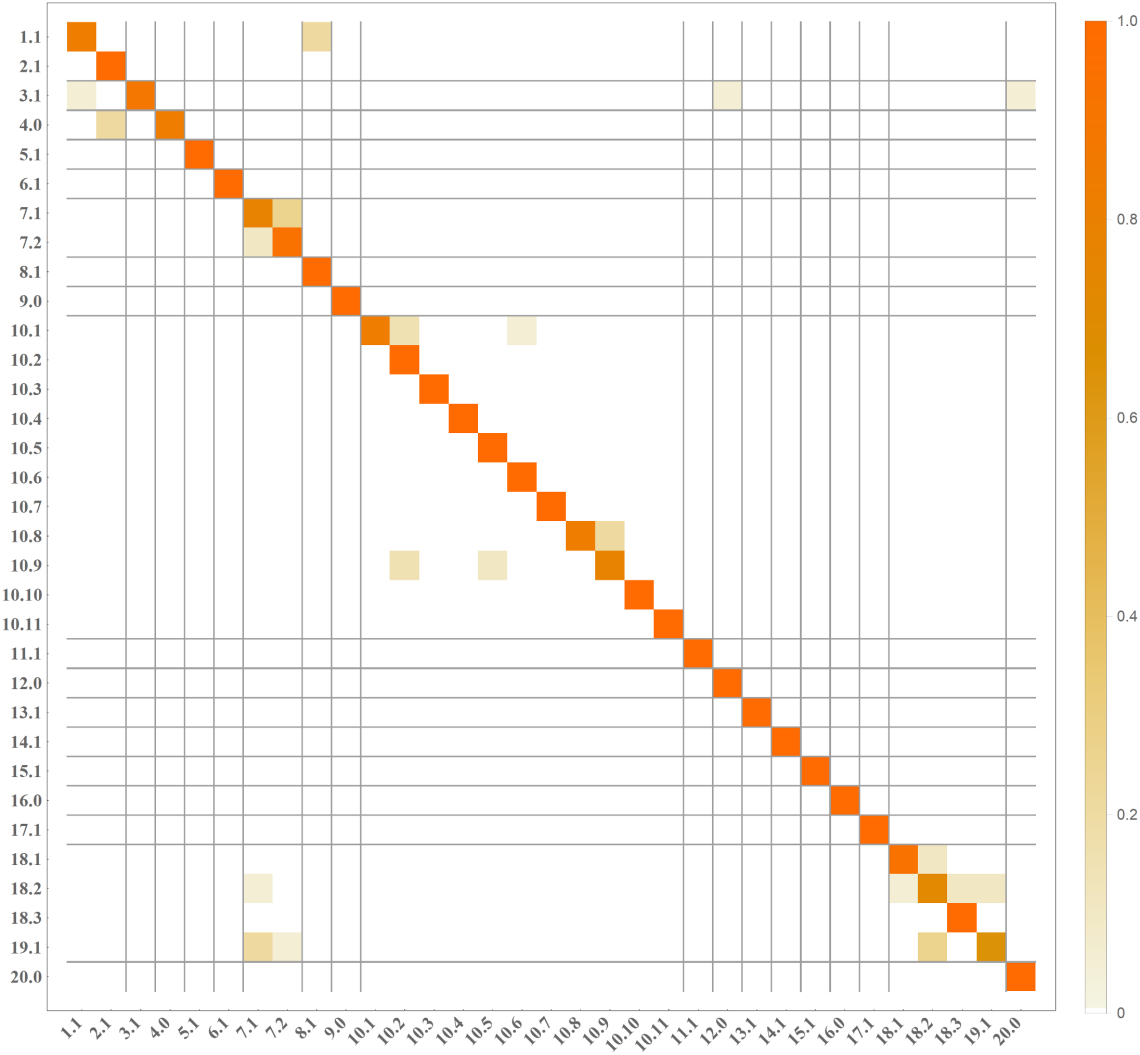
Confusion matrix for method FCFV-M & FV-SIFT (with Original classifier). Position (*i*, *j*) in matrix corresponds to number of observations from class *i* classified as class *j*.

Fig 5 presents the confusion matrix for the best methods FCFV-M & FV-SIFT (with Original classifier), which confirms that the classification error occurs mostly within a genera, while the error between different genera is very rare.

**Fig 6:**
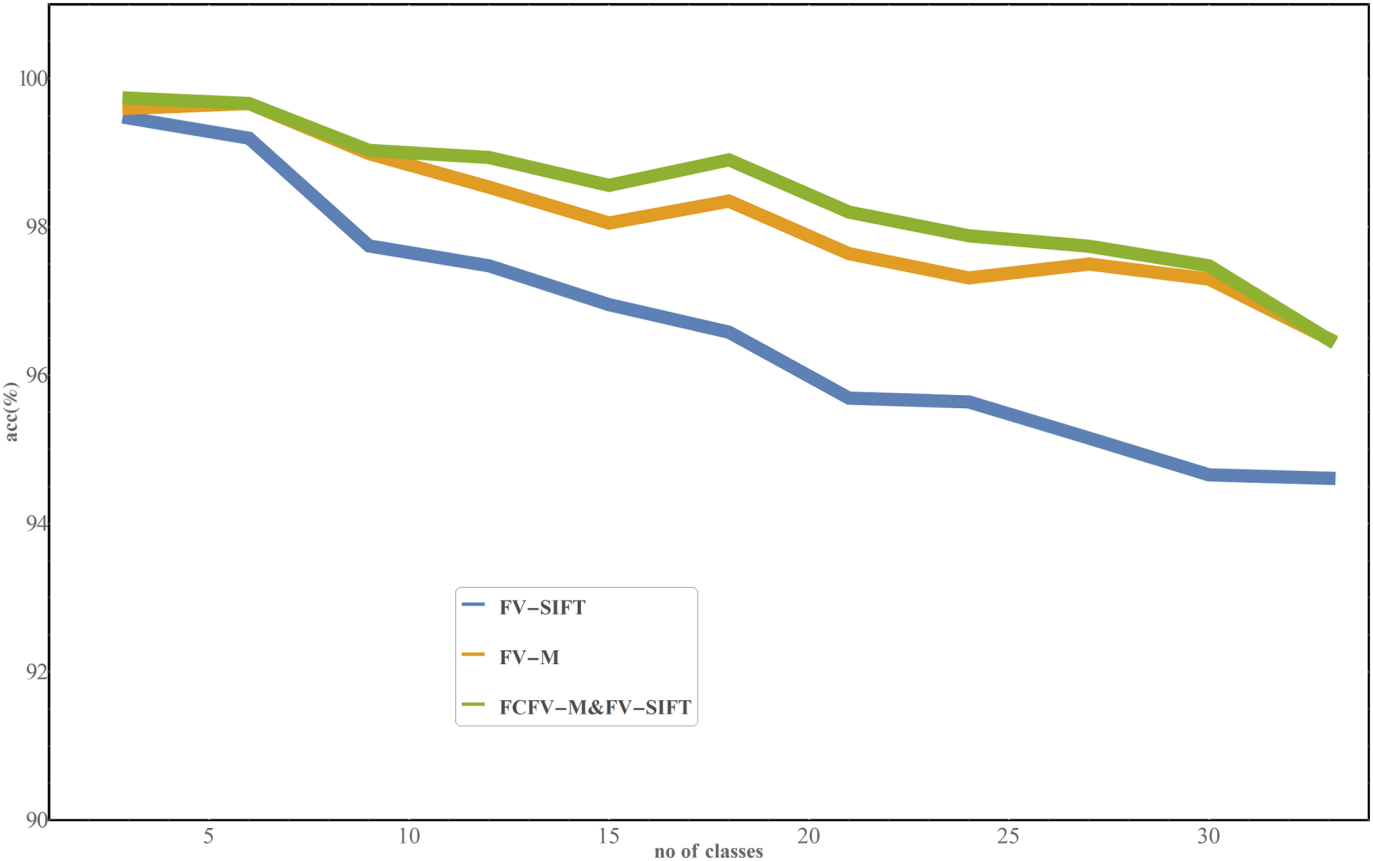
Results of the experiments where we tested the scalability of three most accurate methods: FV-SIFT, FV-M, FCFV-M & FV-SIFT (with Original classifier). The results showed that the accuracy decreases linearly with the number of classes.

*The results of the second experiment* are presented in Fig 6. We can observe that the accuracy of the considered method decreases almost linearly with the number of classes. We performed the classical linear regression and we estimated that the approximation of accuracy for database with 100 classes is:

- 83.42 ±0.34% for FV-SIFT,
- 89.92 ±0.27% for FV-M,
- 90.10 ±0.27% for FCFV-M & FV-SIFT.

This confirms that, the method can be used with decent accuracy, even if the number of classes triples.

## Conclusion

In this paper we apply the state of the art texture recognition method into the problem of classifying bacteria genera and species. To evaluate this approach and to make it comparable with other approaches, we provide a new dataset of images called DIBaS, which is available to other researchers.

According to performed experiments the best approach can be successfully used by the microbiologist in their daily practice, as the accuracy of the recognition is 97.24 ±1.07%. Furthermore, this method can be also used, when database size triples, as the accuracy of the method decreases linearly together with database size.

The future work will concentrate on extending the DIBaS database and on extending the investigated method with the information about the color distribution. This should further improve the accuracy of the recognition.
